# The Predictive Relationship of Health Related Quality of Life on Objectively-Measured Sleep in Children: A Comparison Across BMI Ranges

**DOI:** 10.3389/fnins.2019.01003

**Published:** 2019-09-26

**Authors:** Yi-Ching Lin

**Affiliations:** Department of Early Childhood and Family Education, National Taipei University of Education, Taipei, Taiwan

**Keywords:** children, sleeo, health related quality of life, weight classification, longitudinal study, objective measure

## Abstract

Sleep is considered a major factor related to children’s general quality of life with regards to their health outcomes, general well-being, and daily life functions. Kid-KINDL, a health-related quality of life (HRQOL) measure, was used in a consecutive 12-week longitudinal study to compare the association between children’s quality of life (QoL) and sleep duration across different BMI ranges. To reduce recall bias, each child wore an electronic pedometer on their wrist to record their sleep duration. The Pearson χ2 test, one-way analysis of variance (ANOVA), and mixed effect repeated measures analysis was used to investigate the association between children’s QoL and their sleep duration. The original QoL scores showed that underweight children had lower emotional, family relationship, friendship, and school-related scores, and that overweight children had lower physical satisfaction and self-esteem scores. Emotional (beta = −0.3, *p* < 0.01) and family relationships (beta = 0.20, *p* = 0.01) significantly influenced the sleep duration of underweight children. The score of emotional well-being decreased by 0.3, while sleep duration increased by an hour. The poorer the emotional well-being of children, the longer they slept. The score of family well-being increased by 0.2, while sleep duration increased by an hour. The better family quality children had, the longer they slept; For overweight children, the score of family well-being increased by 0.08, while sleep duration increased by an hour (beta = 0.08, *p* = 0.04). Their sleep got longer when they had better family quality. Physical and school satisfaction scores also significantly affected the sleep duration of obese children. When the score of physical and school increased by 0.09, the sleep duration increases by an hour (beta = 0.09, *p* = 0.01; beta = 0.09, *p* < 0.01, respectively). The better the quality of physical condition and school life was, the longer sleep they would get. This study might be the first longitudinal study to evaluate the relationship between QoL and sleep duration, using an objective device but subjective questionnaires, across BMI ranges in a pediatric population.

## Introduction

Sleep, a crucial physiological need for humans, is important for maintaining health (Lin P.H. et al., 2018), and more specially, sleep duration is mostly used as a sleep variable (Galland et al., 2012). Children from Asian countries are commonly reported to sleep less compared to their counterparts. Sleep duration is a pivotal correlation and factor of various aspects of child development, which relates to quality of life (QoL) in general (El-Sheikh and Kelly, 2017). Sufficient sleep is critical for children’s comprehensive functions: physical health, cognitive processing, and socioemotional adjustment and mental functioning (Paruthi et al., 2016; El-Sheikh and Kelly, 2017; Lin et al., 2018a). The association between sleep duration and QoL has been well discussed in the adult population, as well as in clinical cases in children (Gruber et al., 2014). A longitudinal study indicated that short sleep duration had increased the odds of poor QoL (Roberts et al., 2009) while a cross-sectional study stated that longer sleep duration is associated with better QoL (Do et al., 2013; Perkinson-Gloor et al., 2013).

Health-Related Quality of Life (HRQOL) is an inclusive measurement tool of children’s health outcomes, general well-being, and everyday functions (Delahaye et al., 2014). More specifically, HRQOL has a wide-ranging scope of elements that elaborate how QoL affects children’s physical and psychological condition, social experiences, independence, and environmental influences (Lin, 2018; Lin Y.C. et al., 2018). It is measured in generic and specific conditions. Generic instruments generate a summary of HRQOL, and specific instruments generate data on health problems associated with specific diseases or malfunctions. Generic instruments are used primarily for health profiles of general groups (Guyatt et al., 1993). HRQOL is used to assess generally healthy or chronically ill groups undergoing treatments (Delahaye et al., 2014).

Kid- KINDL is an all-inclusive and multi-dimensional set of generic HRQOL assessments, intended to measure well-being across different health statuses, e.g., overweight and obesity (Hoedjes et al., 2018; Lin, 2018; Lin et al., 2018a).

Childhood overweight and obesity are global health problems that cause cardiovascular disease, diabetes mellitus, hypertension, and adolescent and adult obesity (Brisbois et al., 2012). Overweight and obese children undergo physical and psychosomatic consequences (Puhl and Latner, 2007; Lin et al., 2013). For instance, they might experience social stigma, which impairs their psychological well-being and, ultimately, their QoL (Farhangi et al., 2017; Hoedjes et al., 2018). They are more likely to have impaired HRQOL than their normal-weight counterparts because a high body mass index (BMI) is negatively associated with a poor QoL (Tsiros et al., 2009). How and how much this association affects health outcomes, like poor sleep duration, warrant additional studies.

Although the association between sleep, overweight, and obesity has been well established in adolescents and adults, little is known about the relationship in young children. Prior studies (Cappuccio et al., 2008; Fatima et al., 2015) have claimed an association between poor sleep duration and weight gain in children, but these findings were confounded because of racial or socioeconomic characteristics of the diverse cohorts studied (Lumeng et al., 2007; Lemola et al., 2011; Pakpour et al., 2019).

There are no published multi-dimensional studies on the effects of sleep duration on children in different BMI ranges. Moreover, most sleep studies have used self-rated or parent-rated questionnaires (Chervin et al., 2000; Owens et al., 2000; Sadeh et al., 2000). The data might be biased because of subjective perceptions. Furthermore, for pediatric groups, measuring sleep is quite difficult because parents become less involved with older children’s sleep duration, bedtime routines, and night wakening (Pakpour et al., 2019). Thus, using an electronic pedometer to objectively capture children’s sleep patterns might be helpful (Lin et al., 2018b, 2019).

Because prior research data were acquired primarily from cross-sectional studies, the effects of longitudinal associations are unknown. Thus, we conducted a 12-week longitudinal study to identify and assess sleep patterns in real time.

## Materials and Methods

### Participants: Recruitment and Eligibility

The study was conducted in a community-centered elementary school in New Taipei City, Taiwan, during the second semester of 2017. We recruited 320 parent-child pairs. The eligibility criteria for children were being enrolled in 1st through 5th grades and having no chronic physical, emotional, or intellectual disabilities. Parents had to be between 25 and 50 years old, be the primary caretakers of the child, be interested in promoting the child’s physical activity (PA), and have no chronic physical, emotional, or intellectual disabilities. Subsequently, four classes from each grade (1st through 5th) were randomly selected. All participants were informed of the study purpose and their right to drop out of the study at any time for any reason without prejudice. Signed written informed consent was obtained from all the children’s parents.

The children were asked to wear the waterproof MI Band as often and as long as possible to fully record their sleep duration. The children were issued the user names and passwords for their MI Band and parents authorized the data for use by the research team. Trained research assistants retrieved and recorded the data every 2 weeks. The study procedures were approved by the Institutional Review Board of National Taiwan University Hospital (IRB#: 201604076RIND).

### Measures

#### Anthropometric Measures

Height was measured to the nearest 0.1 cm using a stadiometer, and weight was measured to the nearest 0.01 kg using portable digital scales. From these data, body mass index (BMI) was calculated and subsequently converted to age- and sex-specific standardized scores using the 1990 growth curves from Cole et al. (1995). We used the standard BMI ranges at different age levels from the Taiwan Ministry of Health and Welfare.

#### Health-Related Quality of Life Inventory: Kid-KINDL Proxy

The Kid-KINDL Proxy is a generic QoL instrument designed for parents to rate QoL for their 8- to 13-year-old children. The Proxy has 24 items in six domains: [1]. Physical well-being (4 items; e.g., “My child feels tired and worn-out”); [2]. Emotional well-being (4 items; e.g., “My child feels alone”); [3]. Self-esteem (4 items; e.g., “My child is proud of him-/herself”); [4]. Family (4 items; e.g., “My child gets on well with my parents”); [5]. Friends (4 items; e.g., “My child gets on well with his/her friends”); and [6]. School (4 items; e.g., “My child easily copes with schoolwork”). Each item was rated on a five-point Likert-type scale from “Never” (1) to “all the time” (5), and the scores were converted into a 0–100 scale using (1) = 100, (2) = 75, (3) = 50, (4) = 25, and (5) = 0, where a higher score represents a higher QoL. The construct validity of Kid-KINDL Proxy (Taiwan version) has been tested and confirmed using confirmatory factor analysis (Lin et al., 2014; Lin and Lin, 2017). The internal consistency of the entire set of items was 0.78. The data were collected before the consecutive 12-weeks. The participating parents were asked to fill out the QoL questionnaire and to report the level of each domain for their children.

#### Sleep Duration

A pedometer, the MI Band, was worn on the children’s wrist to detect their PA and sleep duration (Weiss et al., 2010; Meltzer et al., 2012, 2015). Five main health indicators—heart rate, number of steps walked, energy consumption, distance walked, and sleep duration—are the most common indicators used in these devices. Those indicators are validated by determining their respective mean absolute percentage errors (MAPEs). The MI Band is highly accurate: MAPE = ca. 0.10 (Shelgikar et al., 2016; Xie et al., 2018). Data were extracted on at least three weeknights and at least one weekend night (for weekend period) for calculation.

The study started from the Fall semester in 2017. Children were asked to wear the MI Band for 12 consecutive weeks for the purpose of research data collection. Their averaged sleep duration from weekdays and weekends were both retrieved by research assistants and then generated for the study. However, compared to children’s sleep duration during weekends, their sleep duration during weekdays was more confined by the school schedule. Weekday data were more consistent, rather than the weekend data that may include the need of “catch-up” sleep (Epstein et al., 1998). Therefore, considering their actual bodily needs and sleep habits, the data of their sleep duration on weekends was also considered for the analysis.

### Statistical Analysis

The two major objectives of the study were the effects of QoL on sleep duration and how they compared across different BMI ranges (underweight, normal weight, overweight and obesity). Significance was set at *p* < 0.05. The analyses were conducted through bivariate associations between the studied variables using cross tabulations and one-way ANOVA. Moreover, a mixed effect repeated measures analysis was performed to estimate children’s sleep. The associations between children’s QoL, defined as a fixed effect, and children’s sleep duration, in four different linear mixed effects models with different BMI ranges, were measured through a 12-week period. The model tested the divergence of sleep duration based on children’s QoL to respond to the research assumptions. The analyses described above employed the Statistical Package for Social Sciences (SPSS) statistical software version 20.0 package (IBM Corp., Armonk, NY).

## Results

We assessed the data of 263 children (121 [46%] were boys; 184 [ca. 70%] were normal-weight, and 65 [ca. 25%] were overweight and obese (Table 1). The effect of QoL on sleep duration on weekdays, weekends, and entire weeks for children were all examined as a sensitivity analysis (Appendix A). The results showed that children’s QoL could not significantly predict their sleep duration on weekdays, but, for obese children, their school domain in QoL had a significantly positive impact on their sleep duration. In other words, the better their experience in school, their longer they sleep. As aforementioned, only the sleep duration on weekends was further explored and the findings were presented as follows: For different aspects of heath related QoL, the school domain had the lowest mean (64.12), and the physical domain had the highest (74.62); Underweight children scored low on the Emotion, Family, Friend, and School domains, while obese children scored high on the Family, Friend, and Physical domains (Table 1).

**TABLE 1:** Descriptive statistics for participants’ background characteristics and Kid-KINDL Quality of Life across different BMI ranges.

		**All**	**Underweight 14(5.3%)**	**Normal 184(70%)**	**Overweight 40(15.2%)**	**Obese 25(9.5%)**	***X*^2^ or F**	***P***
**Sex**							0.68	0.88
	Boys	121(46%)	5(35.7%)	85(46.2%)	19(47.5%)	12(48%)		
	Girls	142(54%)	9(64.3%)	99(53.8%)	21(52.5%)	13(52%)		
**Grade**							11.28	0.51
	1st	52(19.8%)	3(5.77%)	34(65.38%)	13(25.00%)	2(3.85%)		
	2nd	68(25.9%)	4(5.88%)	49(72.06%)	8(11.76%)	7(10.29%)		
	3th	48(18.3%)	4(8.33%)	35(72.92%)	6(12.50%)	3(6.25%)		
	4th	44(16.7%)	2(4.55%)	30(86.18%)	7(15.91%)	5(11.36%)		
	5th	51(19.4%)	1(1.96)	36(70.59%)	6(11.76%)	8(15.69%)		
**BMI**		17.42 ± 3.01	13.57 ± 0.65	16.28 ± 1.49	19.88 ± 1.47	24.08 ± 1.98		
**Kid-KINDL QoL**								
Physical		74.62 ± 15.30	72.60 ± 11.56	75.21 ± 15.81	71.53 ± 13.65	76.04 ± 15.71	0.72	0.54
Emotion		74.21 ± 16.32	71.15 ± 12.90	74.20 ± 16.89	75 ± 13.74	74.73 ± 18.02	0.19	0.91
Esteem		68.96 ± 18.92	73.08 ± 16.81	69.34 ± 19.57	66.39 ± 17.82	68 ± 17.15	0.48	0.70
Family		71.87 ± 16.23	67.31 ± 15.34	70.93 ± 16.59	75 ± 13.66	76.25 ± 16.93	1.62	0.19
Friend		70.11 ± 17.76	62.97 ± 15.96	70.04 ± 17.92	71.34 ± 17.54	72.75 ± 17.85	1.00	0.39
School		64.12 ± 17.33	54.91 ± 20.54	65.60 ± 17.34	62.33 ± 14.47	61.50 ± 18.10	2.10	0.10
Total		70.80 ± 11.75	66.83 ± 9.33	71.01 ± 12.37	70.39 ± 9.491	72.10 ± 11.67	0.62	0.60

Mixed-effect repeated measures measured that sex, age, and BMI were not significant for sleep but that the length of the study negatively affected all of the participants’ sleep. The consecutive weeks seemed like an important factor that impacted the children’s sleep (Table 2). The findings showed that on-going weekends had a significant effect on children’s sleep for children of underweight, normal weight, overweight and obese groups respectively [*F*(1, 25.04) = 7.47^∗∗^; *F*(1, 632.08) = 77.78^∗∗∗^; *F*(1, 127.81) = 15.328^∗∗∗^; *F*(1, 79.51) = 15.42^∗∗∗^]. As the study went on (β ≤ −0.35, *p* ≤ 0.01), the worse their sleep became, regardless of their BMI ranges.

**TABLE 2:** Health-related quality of life indicators of children’s sleep duration across different BMI ranges.

	**Underweight**			**Normal Weight**			**Overweight**			**Obese**		
**Variables**	**β**	**SE**	***p***	**β**	**SE**	***p***	**β**	**SE**	***p***	**β**	**SE**	***p***
Intercept	35.83	9.86	0.00	6.72	1.93	0.00	–1.28	4.05	0.75	–12.9	7.58	0.10
Sex	2.55	1.38	0.09	0.59	0.37	0.11	1.49	0.83	0.08	**1.81**	**0.83**	**0.03**
Age	0.71	0.75	0.36	0.00	0.00	0.59	0.34	0.36	0.34	0.00	0.00	0.39
Week	−**0.30**	**0.11**	**0.01**	−**0.35**	**0.04**	**0.00**	−**0.31**	**0**.**08**	**0.00**	−**0.27**	**0.07**	**0.00**
BMI	–2.21	1.01	0.05	−0.12	0.10	0.22	–0.21	0.25	0.40	0.20	0.13	0.14
**Kid-KINDL QoL**												
Physical	–0.07	0.07	0.31	0.02	0.01	0.22	0.00	0.04	0.94	**0.09**	**0.03**	**0.01**
Emotion	−**0.30**	**0.07**	**< 0.01**	0.00	0.01	0.87	0.02	0.04	0.57	0.03	0.03	0.42
Esteem	0.08	0.06	0.21	0.00	0.01	0.89	–0.02	0.03	0.36	0.02	0.03	0.31
Family	**0.20**	**0.06**	**0.01**	0.00	0.01	0.96	**0.08**	**0.04**	**0.04**	–0.02	0.02	0.17
Friend	0.03	0.06	0.59	−0.02	0.01	0.21	–0.02	0.02	0.50	–0.05	0.04	0.11
School	–0.03	0.03	0.34	0.02	0.01	0.20	0.04	0.03	0.18	**0.09**	**0.02**	**< 0.01**
**Interaction between QoL with Weekend Sleep^*a*^**												
Physical	0.00	0.03	0.90	–	–	–	0.00	0.05	0.94	0.03	0.04	0.42
Emotion	–0.07	0.05	0.12	–	–	–	–0.08	0.05	0.15	–0.04	0.04	0.36
Esteem	0.05	0.04	0.22	–	–	–	0.00	0.04	0.98	0.05	0.05	0.28
Family	0.03	0.04	0.46	–	–	–	0.05	0.04	0.14	–0.05	0.03	0.11
Friend	0.00	0.04	0.90	–	–	–	0.00	0.03	0.95	–0.02	0.06	0.76
School	0.00	0.03	0.96	–	–	–	0.02	0.03	0.52	0.04	0.03	0.22

*^*a*^For the interaction models between QoL with Weekend Sleep, the normal weight group was used as the reference group. Bold values indicate being statistically significant at level of *p* < 0.05.*

For groups of underweight children, the emotional well-being [*F*(1, 12.78) = 16.18^∗∗^] and family [*F*(1, 12.78) = 9.36^∗∗^] dimensions were potentially important predictors of children’s sleep. The effect of emotional well-being was significantly but negatively associated with children’s sleep duration, which indicated when the score of emotional well-being decreased by 0.3, the sleep duration increased by an hour (beta = −0.3, *p* < 0.01). The poorer the emotional well-being of children, the longer they slept. In addition, the effect of family was significant, and its coefficient was positive when the score of family well-being increased by 0.2, while sleep duration increased by an hour (beta = 0.20, *p* = 0.01). In other words, the better family quality children had, the longer they slept.

On the other hand, for the group of overweight children, family is a possibly critical predictor of their sleep [*F*(1, 59.07) = 3.85^∗∗^]. The effect of family was significant, and its coefficient showed when the score of family well-being increased by 0.08, the sleep duration increased by an hour (beta = 0.08, *p* = 0.04). For overweight children, their sleep duration became longer when they had better family quality. In addition, for the group of obese children, physical condition [*F*(1, 40.46) = 7.59^∗∗^] and school [*F*(1, 40.46) = 10.98^∗∗^] were possibly critical factors of their sleep. The effect of physical condition and school were significant, and their coefficients were positive when the physical condition and school score increased by 0.09, while sleep duration increased by an hour (beta = 0.09, *p* = 0.01; beta = 0.09, *p* < 0.01, respectively). The better the quality of physical condition and school life was, the longer the duration of sleep they would get. However, there was no statistical significance between QoL and the sleep of children with a normal weight, which means that these dimensions of QoL were not significant factors associated with the sleep of children of a normal weight.

In order to further look into whether the association between QoL and sleep duration differs by BMI ranges, the interaction models were applied using the normal weight group as the reference group. The results indicated no significant interactions, which showed that the influences of QoL on sleep duration were not different between children of a normal weight and their counterparts.

## Discussion

Given a lack of studies on the association between children’s QoL and their sleep duration (Chaput et al., 2016), the findings are expected to fulfill the research gap. The present study found that, over the course of the semester, children’s QoL had a significantly negative effect on sleep duration, which is opposite to the literature (Liguori et al., 2011; Do et al., 2013; Perkinson-Gloor et al., 2013). A possible explanation could be that, the levels of the QoL, for the children that participated, were identified at the beginning of the semester, but their sleep duration were measured as the semester proceeded. Usually, school load increases as the semester continues; therefore, the sleep duration could decrease along with the consecutive weeks.

Other important findings were that underweight children had lower scores in the emotion, family, friends, and school domains, and that overweight children had lower scores in the physical and self-esteem domains. These findings were consistent with prior studies (e.g., Friedlander et al., 2003; Sato et al., 2008; Strong et al., 2017). We also found that, for underweight and overweight children, the quality of their family life significantly affected their sleep duration, which is consistent with a prior study (Lumeng et al., 2007) that reported poor sleep duration and being overweight might simply reflect an underlying lack of daily life structure or unsatisfying parenting in the family. Children’s sleep patterns are embedded and shaped by family relationships (El-Sheikh and Kelly, 2017), however, children’s sleep duration within a family context is usually investigated as a correlation, rather than being investigated by familial factors through longitudinal studies (Kelly and El-Sheikh, 2011).

Although the developmental trajectories of children’s emotional (internalized) problems have often been addressed, limited attention has been given to underweight children. Underweight children are more likely to have internalized problems (e.g., depression and anxiety) than normal-weight and overweight children are (Cimino et al., 2016), for girls in particular (Xie et al., 2013). For underweight children, the present study found that the worse the emotional wellbeing was, the longer they slept, which is consistent with prior literature (Wolfson and Carskadon, 1998; Yen et al., 2010) indicating that suboptimal emotion is related to longer sleep duration. It is also assumed that children may use sleep as a get-away approach to avoid emotional difficulties in real life. However, limited literature and the present finding emphasize the need for future research on the relationship between emotional problems and long sleep duration. We also found that the physical and school domains significantly affected the sleep duration of obese children: the higher their scores in these domains were, the longer they slept. This is consistent with prior studies which claim that, compared with normal-weight children, overweight and obese children are two to four times more likely to have a higher risk of physical functioning and psychosocial health-related internalized problems (Friedlander et al., 2003; Dumuid et al., 2017). Therefore, it is understandable that children’s sleep duration could become longer if they are in optimal physical and psychosocial conditions; the phenomenon was significant for obese children because they were more likely to encounter discrimination (social stigma) or social isolation due to their oversized bodies (Lin and Lin, 2017). If they feel positive about their physical self-perception and social interaction in school, the experiences will promote their sleep duration.

This seems to be the first longitudinal study that has used an objective device, a MI-Band pedometer worn on the wrist, to objectively record and evaluate sleep duration and its relationship with HRQOL across BMI ranges in elementary school children. The significance of our findings is that we used an objective measure in addition to subjective questionnaires to avoid bias from retrospective memories. The wrist-pedometer has become popular for detecting the sleep patterns of children because it is inexpensive, portable, and user friendly (Meltzer et al., 2012, 2015). Additionally, using pedometers to assess sleep duration gives an advantage for the area of inquiry, which allows multiple sleep parameters to be measured simultaneously without recall bias (Kouros and El-Sheikh, 2017). This study also showed the integrated and subscale effects of domains on children’s sleep. We identified unique information distinct from physiological measures using Kid-KINDL. These findings should lead to research that is even more innovative.

### Limitations

Our study had limitations. First, the study was done in an urban city with a small sample of 263 parent-child dyads, which might limit the generalizability of the findings. Second, although the proxy report seems reasonable when children are too young to respond on their own (Lin Y.C. et al., 2018), the bias from a proxy report is inevitable: e.g., parents of overweight and obese children are more likely to report their children’s health and well-being as poor. This bias needs to be recognized and understood. Additionally, the data of health-related QoL was not collected every week with the sleep duration. The solidity of the analyses would have been strengthened if the levels of QoL were collected every week or at least more frequently.

## Conclusion

The study used repeated and objective assessments of children’s sleep duration, underlining the necessity of consistent efforts to promote sufficient sleep for children’s overall health. Children’s QoL might be a feasible and accurate indicator of their sleep duration and quality. Even though the results did not differ by BMI ranges, the findings can be perceived as a preventive health promotion approach for school-aged children. More studies that relate to the association between children’s QoL and sleep are warranted.

## Data Availability

All datasets generated for this study are included in the manuscript and/or the supplementary files.

## ETHICS Statement

This study was carried out in accordance with the recommendations of the research ethics guidelines from the Institutional Review Board (IRB) in the University of Taipei with written informed consent from all subjects. All subjects gave written informed consent in accordance with the Declaration of Helsinki. The protocol was approved by the Institutional Review Board (IRB; approval no. 201604076RIND) in the University of Taipei.

## Author Contributions

Y-CL conceptualized the structure of the study, collected the data, conducted the formal analysis, wrote the manuscript, and completed the final review and editing solely.

## Conflict of Interest Statement

The author declares that the research was conducted in the absence of any commercial or financial relationships that could be construed as a potential conflict of interest. The handling Editor, C-YL declared a past co-authorship with the author, Y-CL.

## Appendix

**APPENDIX A:** Health-related quality of life indicators of children’s sleep duration across different BMI ranges in consecutive 12 weekdays, weekends and weeks.

	**Underweight**									**Normal weight**									**Overweight**									**Obese**								
	**Weekdays**			**Weekends**			**Weeks**			**Weekdays**			**Weekends**			**Weeks**			**Weekdays**			**Weekends**			**Weeks**			**Weekdays**			**Weekends**			**Weeks**		
	***B***	**SE**	***p***	***B***	**SE**	***p***	***B***	**SE**	***p***	***B***	**SE**	***p***	***B***	**SE**	***p***	***B***	**SE**	***p***	***B***	**SE**	***p***	***B***	**SE**	***p***	***B***	**SE**	***p***	***B***	**SE**	***p***	***B***	**SE**	***p***	***B***	**SE**	***p***
Intercept	−2.94	4.83	0.56	0.87	3.09	0.78	−0.85	4.57	0.86	−0.22	0.09	0.01	−0.20	0.07	0.00	−0.25	0.08	0.00	1.26	2.27	0.58	1.38	1.85	0.46	1.41	2.23	0.53	−0.66	0.44	0.14	−0.72	0.38	0.06	−0.77	0.45	0.10
Sex	0.70	0.51	0.22	0.60	0.33	0.09	0.65	0.48	0.22	0.09	0.11	0.39	0.14	0.09	0.11	0.11	0.10	0.30	0.37	0.24	0.14	0.35	0.20	0.08	0.37	0.24	0.12	0.60	0.23	0.01	**0.43**	**0.20**	**0.03**	0.52	0.23	0.03
Age	−5.24	39.16	0.90	23.64	25.07	0.36	11.88	37.07	0.76	0.09	0.09	0.35	0.04	0.08	0.59	0.06	0.09	0.54	11.20	14.63	0.45	11.49	11.92	0.34	12.23	14.38	0.40	0.05	0.10	0.58	0.07	0.08	0.39	0.06	0.10	0.56
Week	**−0.20**	**0.12**	**0.01**	**−0.25**	**0.09**	**0.01**	**−0.25**	**0.11**	**0.04**	**−0.22**	**0.03**	**0.00**	**−0.29**	**0.03**	**<0.01**	**−0.28**	**0.03**	**<0.01**	**−0.18**	**0.07**	**0.01**	**−0.26**	**0.07**	**<0.01**	**−0.24**	**0.07**	**<0.01**	**−0.17**	**0.08**	**0.04**	**−0.29**	**0.07**	**<0.01**	**−0.26**	**0.08**	**<0.01**
BMI	−1.40	1.34	0.34	**−1.87**	**0.86**	**0.05**	−1.78	1.27	0.21	−0.03	0.10	0.78	−0.11	0.09	0.22	−0.07	0.10	0.50	−0.19	0.26	0.47	−0.18	0.21	0.40	−0.20	0.26	0.45	0.16	0.18	0.38	0.24	0.16	0.14	0.21	0.19	0.26
** Kid-KINDL QoL**																		
Physical	−0.42	0.39	0.32	−0.26	0.25	0.31	−0.32	0.37	0.42	0.03	0.06	0.55	0.06	0.05	0.22	0.05	0.06	0.37	−0.01	0.17	0.98	0.01	0.14	0.94	0.01	0.17	0.93	0.38	0.16	0.02	**0.37**	**0.14**	**0.01**	**0.39**	**0.16**	**0.02**
Emotion	−1.09	0.45	0.05	**−1.15**	**0.29**	**<0.01**	**−1.09**	**0.42**	**0.04**	0.02	0.06	0.70	0.01	0.05	0.87	0.01	0.06	0.84	−0.03	0.19	0.90	0.09	0.16	0.57	0.03	0.19	0.87	0.07	0.17	0.68	0.12	0.15	0.42	0.11	0.17	0.54
Esteem	0.29	0.39	0.49	0.33	0.25	0.21	0.28	0.37	0.47	0.02	0.06	0.80	0.01	0.05	0.89	0.01	0.06	0.82	−0.08	0.14	0.56	−0.11	0.11	0.36	−0.10	0.14	0.46	0.29	0.24	0.24	0.22	0.21	0.31	0.27	0.25	0.28
Family	0.56	0.38	0.19	**0.75**	**0.25**	**0.01**	0.67	0.36	0.11	0.00	0.06	0.97	0.00	0.05	0.96	0.00	0.06	0.95	0.32	0.18	0.08	**0.29**	**0.15**	**0.05**	0.31	0.18	0.08	−0.17	0.13	0.21	−0.16	0.12	0.17	−0.18	0.14	0.21
Friend	0.29	0.38	0.48	0.14	0.25	0.59	0.17	0.36	0.66	−0.09	0.07	0.22	−0.07	0.06	0.21	−0.08	0.07	0.24	0.07	0.11	0.52	−0.06	0.09	0.50	0.00	0.11	0.98	−0.46	0.25	0.08	−0.35	0.22	0.11	−0.42	0.26	0.11
School	0.00	0.17	0.99	−0.11	0.11	0.34	−0.05	0.16	0.77	0.07	0.06	0.25	0.07	0.05	0.20	0.07	0.06	0.25	0.09	0.14	0.54	0.15	0.11	0.18	0.13	0.14	0.34	**0.50**	**0.16**	**<0.01**	**0.45**	**0.13**	**<0.01**	**0.48**	**0.16**	**0.01**

*Bold values indicate being statistically significant at level of *p* < 0.05.*
